# P084 Beyond headache: an atypical relapse of giant cell arteritis presenting with fatigue and dizziness

**DOI:** 10.1093/rap/rkag113.114

**Published:** 2026-11-03

**Authors:** Mohammed Shugaa Addin, Rehan Asif

**Affiliations:** Aneurin Bevan University Health Board, Newport, United Kingdom; Aneurin Bevan University Health Board, Newport, United Kingdom

## Abstract

**Introduction:**

This case describes a 73-year-old woman with relapsing giant cell arteritis (GCA) who initially presented with headaches and raised CRP, responded promptly to corticosteroids, but subsequently developed recurrent inflammatory activity during steroid tapering. Despite a normal temporal artery ultrasound and initially stable disease control, she later developed biochemical relapse, PMR-type symptoms, and ultimately PET-CT evidence of active large vessel vasculitis (LVV) while on leflunomide.

This highlights diagnostic uncertainty; the limited value of ultrasound in steroid-pre-treated patients; evolving disease phenotype; and the challenge of balancing steroid toxicity, DMARD efficacy, and timely escalation to biologic therapy.

**Case description:**

A 73-year-old woman presented in November 2024 with two weeks of left-sided neck pain, frontal headaches, and bilateral temporal tenderness. CRP was 44 mg/L with a normal ESR. She had no visual symptoms, jaw claudication, fevers, or systemic features. Prednisolone 40 mg daily was commenced, resulting in complete resolution of headaches within 48 hours. A temporal artery ultrasound performed several days later was normal. She was managed as probable GCA and commenced a structured taper.

By January 2025, she was stable on 15 mg of prednisolone with a normal CRP and no GCA symptoms. However, during further tapering to 7 mg she developed recurrent headaches and rising CRP (38–62 mg/L). Prednisolone was increased to 20 mg with rapid symptomatic improvement. Avoiding another relapse, leflunomide 20 mg daily was initiated in July 2025. CRP normalised, and she successfully tapered to 12.5 mg, then 8 mg prednisolone by November 2025.

In early 2026 she remained clinically stable on 5 mg prednisolone, though mildly fatigued. CRP rose gradually from 12 to 24 mg/L. By May 2026, after tapering to 3 mg prednisolone, she developed increased dizziness, fatigue, and generalised stiffness without headaches or cranial symptoms. CRP increased to 36 mg/L. Examination showed no temporal tenderness, synovitis, or bruits. PMR was suspected, and PET-CT was arranged.

PET-CT (June 2026) demonstrated active LVV involving the aorta and bilateral subclavian arteries, with additional PMR-pattern uptake in shoulders and pelvic girdle. Findings indicated persistent vasculitis despite leflunomide. Leflunomide was discontinued and screening initiated for biologic therapy. Tocilizumab was recommended, with interim prednisolone escalation to 10 mg.

DEXA scanning showed normal bone density despite long-term steroid exposure. She remains under close monitoring pending biologic initiation.

**Discussion:**

This case illustrates several challenges in managing GCA, particularly relapse during steroid tapering, diagnostic uncertainty following early steroid exposure, and evolution toward PMR and LVV phenotypes. Her initial presentation was typical for cranial GCA, with rapid steroid response and raised CRP. However, temporal artery ultrasound was performed after several days of high-dose corticosteroids, reducing sensitivity. This emphasises the need for imagining early if suspecting and the limitations of ultrasound in steroid-pre-treated patients.

Her subsequent disease course demonstrated biochemical relapse and recurrent headaches during tapering, consistent with steroid-responsive GCA. Introducing leflunomide provided partial control, allowing tapering to low-dose prednisolone. However, rising CRP and development of PMR-type symptoms suggested evolving systemic inflammation. PMR and LVV frequently coexist with GCA, and the emergence of proximal limb stiffness in this context warranted further evaluation.

PET-CT was decisive. It demonstrated active inflammation in the aorta and subclavian arteries, confirming LVV despite DMARD therapy. PET-CT also identified PMR-pattern uptake, supporting the clinical picture. This case highlights the value of PET-CT in assessing atypical or relapsing disease, particularly when inflammatory markers rise without cranial symptoms. PET-CT is increasingly recognised as a key modality for diagnosing LVV and monitoring complex GCA phenotypes.

Treatment decisions were shaped by competing risks, including steroid toxicity, hypertension, fatigue, and the need for effective disease control. Leflunomide was insufficient, and biologic therapy with tocilizumab was appropriately selected. Tocilizumab is effective for both cranial and large-vessel GCA and facilitates steroid minimisation. Interim prednisolone escalation was required to control symptoms while awaiting biologic initiation.

This case raises important questions regarding optimal DMARD selection, timing of biologic therapy, and the role of PET-CT in monitoring relapse. It also emphasises the need for multidisciplinary coordination and patient-centred decision-making in long-term GCA management.

**Key learning points:**

1. Dizziness can be an atypical manifestation of GCA

Although classical cranial symptoms dominate, GCA may present with nonspecific neurological symptoms such as dizziness or presyncope, particularly when extracranial carotid or vertebral vessels are involved. These symptoms are easily misattributed to cardiovascular or autonomic causes, delaying diagnosis (Salvarani et al., 2008; Kermani et al., 2015).

2. Relapse during steroid tapering is common and requires vigilant monitoring

Rising CRP or recurrence of headaches during tapering should prompt reassessment of disease activity. Inflammatory markers alone are insufficient, and clinical evaluation—including screening for PMR symptoms—is essential (Buttgereit et al., 2018; Hellmich et al., 2020).

3. PET-CT is invaluable in atypical or relapsing disease

PET-CT provides diagnostic clarity when symptoms evolve or inflammatory markers rise without cranial features. It can identify large-vessel involvement and PMR-pattern uptake, guiding escalation to biologic therapy. Optimal interpretation requires patients to be on very low-dose steroids or ideally off steroids, as glucocorticoids rapidly suppress FDG uptake (Prieto-González et al., 2014; Slart et al., 2018).

